# An injury mortality prediction based on the anatomic injury scale

**DOI:** 10.1097/MD.0000000000007945

**Published:** 2017-09-01

**Authors:** Muding Wang, Dan Wu, Wusi Qiu, Weimi Wang, Yunji Zeng, Yi Shen

**Affiliations:** a Department of Emergency Medicine, Affiliated Hospital of Hangzhou Normal University; b Department of Surgery, The Second Affiliated Hospital of Zhejiang University School of Medicine; c Department of Neurosurgery, Affiliated Hospital of Hangzhou Normal University; d Department of Orthopedic, Affiliated Hospital of Hangzhou Normal University; e Department of Epidemiology and Health Statistics, School of Public Health, Zhejiang University, Hangzhou, Zhejiang, China.

**Keywords:** abbreviated injury scale, injury mortality prediction, prediction of mortality, trauma mortality prediction model

## Abstract

To determine whether the injury mortality prediction (IMP) statistically outperforms the trauma mortality prediction model (TMPM) as a predictor of mortality.

The TMPM is currently the best trauma score method, which is based on the anatomic injury. Its ability of mortality prediction is superior to the injury severity score (ISS) and to the new injury severity score (NISS). However, despite its statistical significance, the predictive power of TMPM needs to be further improved.

Retrospective cohort study is based on the data of 1,148,359 injured patients in the National Trauma Data Bank hospitalized from 2010 to 2011. Sixty percent of the data was used to derive an empiric measure of severity of different Abbreviated Injury Scale predot codes by taking the weighted average death probabilities of trauma patients. Twenty percent of the data was used to create computing method of the IMP model. The remaining 20% of the data was used to evaluate the statistical performance of IMP and then be compared with the TMPM and the single worst injury by examining area under the receiver operating characteristic curve (ROC), the Hosmer–Lemeshow (HL) statistic, and the Akaike information criterion.

IMP exhibits significantly both better discrimination (ROC-IMP, 0.903 [0.899–0.907] and ROC-TMPM, 0.890 [0.886–0.895]) and calibration (HL-IMP, 9.9 [4.4–14.7] and HL-TMPM, 197 [143–248]) compared with TMPM. All models show slight changes after the extension of age, gender, and mechanism of injury, but the extended IMP still dominated TMPM in every performance.

The IMP has slight improvement in discrimination and calibration compared with the TMPM and can accurately predict mortality. Therefore, we consider it as a new feasible scoring method in trauma research.

## Abstract 2

Supplemental Digital Content is available in the text

## 1 Introduction

The trauma score has an independent score method after the establishment of the Abbreviated Injury Scale (AIS).^[1]^ It is the one that is designed by the only dictionary especially as a system to define the severity of injuries through the body. The latest version of such technique appeared in 2005,^[2]^ which was updated in 2008.^[3]^ Although the injury severity score (ISS)^[4]^ of AIS-based severity values has been considered as a “gold standard” in the anatomic injury severity indicators and widely used in clinical science, the real definition is a “sum of squares,” which is possible to lose part of the calculating information of trauma and lead to the poor ability of predicting mortality.^[5–8]^ The new injury severity score (NISS)^[5]^ overcomes the shortcoming of ISS, but overestimates the mortality of injuries in the same body region (BR), so it cannot completely replace the ISS.^[9]^ The logarithm injury severity score (LISS)^[6]^ adopts natural logarithm (ln) to transform AIS and automatically removes the score of 1 or 2 from the AIS, which is better than the NISS and ISS in predicting mortality. However, it is similar to ISS and NISS, which cannot solve the problem that the same value in different BRs does not imply the same mortality rate.^[7,9–11]^

The trauma mortality prediction model (TMPM)^[7]^ derives an empirical severity value for each diverse AIS predot codes, which is called the model-averaged regression coefficient (MARC). Then we calculate the TMPM value according to MARC values and predict the mortality. The TMPM is better than the ISS in discriminating survivors from nonsurvivors. Recently, researchers concluded that the TMPM outperforms the NISS and ISS as a predictor of mortality.^[8]^ Although TMPM is statistically rigorous, it is not accurate enough in mathematics.

We suggest a new method in predicting death of injured patients with the injury severity that is indicated by the AIS code dictionary to fix the inaccuracy of AIS severity measurements, especially the prediction results of TMPM. Based on the National Trauma Data Bank (NTDB) database, first, 60% of the data is used to derive a weighted average death probability (WADP) for each of different AIS predot codes. These WADP values provide a platform for comparing the personal injury severity and are included in the mortality prediction model about personal injury. Then, 20% of the data is used to evaluate the injury mortality prediction (IMP) model (consists of the 5 worst injuries). Last, the remaining 20% of the data (validation data set) is used to compare these 2 new models with the TMPM model based on the latest standard in using measures of discrimination, calibration, and the AIC.

## 2 Methods

### 2.1 Data source

**Figure 1:**
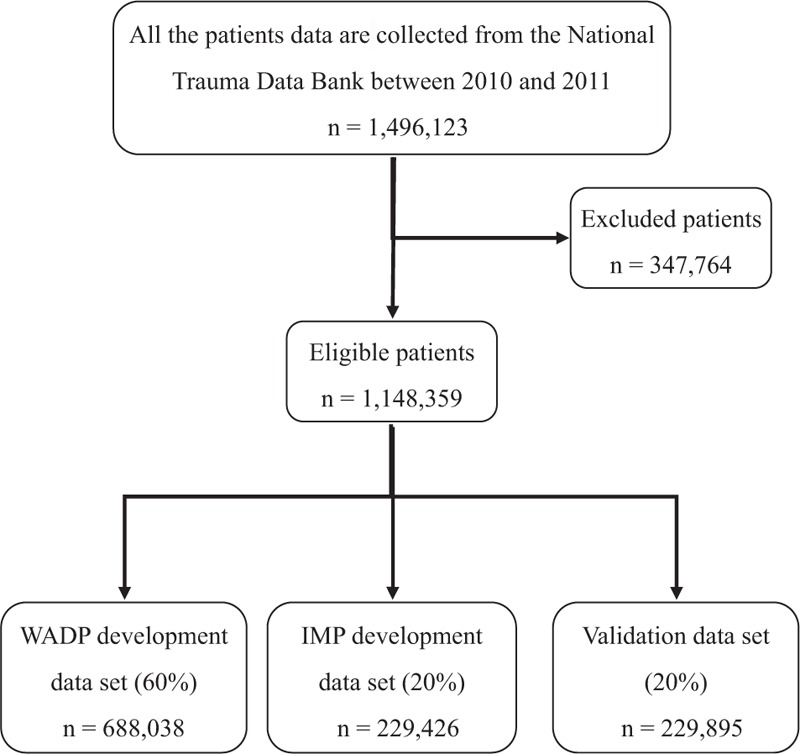
Flowchart for data analyzed. IMP = injury mortality prediction, WADP = weighted average death probability.

This study was conducted using data from the NTDB on patients hospitalized with traumatic injuries between 2010 and 2011. Available information included patient demographics, hospital demographics, AIS codes (version 1998), Glasgow Coma Score (GCS), mechanism of injury (based on International Classification of Diseases, 9th Revision, Clinical Modification External cause of injury codes), encrypted hospital identifiers, and in-hospital mortality. The data set consisted of 1,496,123 patients with one or more AIS codes. Patients without AIS codes (12,301) or with burns or nontraumatic diagnoses (e.g., poisoning, drowning, suffocation) (110,630), with missing or invalid data (data missing on age, sex, length of hospital stay, or outcome) (17,295), and patients who sustained a single injury and AIS code component was 9 (3445), whose age younger than 1 year (85,327), or over 89 years (46,128) were excluded from our analysis. Patients who were dead on arrival (4771) or transferred to another facility (45,396) were also excluded. We also required the hospital to have hospitalized at least 500 trauma patients in 1 year, because we believed that hospitals have substantial experience in trauma treatment, and the AIS coding would be more accurate (107,790 patients were excluded). E-codes were mapped to 1 of the 6 mechanisms of injury by an experienced clinical trauma surgeon: low fall, motor vehicle crash, violence, blunt injury, stab wound, and gunshot wound. The hospital demographics show that the majority of the hospitals in the study data set are Level I and II trauma centers with more than 200 beds and <2000 patients each year, are nonprofit, and are community hospitals and university affiliated hospitals. The final data set included 1,148,359 patients admitted to 476 hospitals. The details for recruitment were shown in Fig. 1.

### 2.2 Overview of IMP development

In our study data set of 1336 AIS injury codes, we evaluated only 60%, lost 7 AIS injury codes (only contains 12 BRs). Therefore, there were 1329 AIS injury codes involved in development of trauma mortality rate (TMR) and WADP eventually. First, we calculated the TMR of each AIS predot code (Appendix A). Then we created 3 separate logistic regression models based on TMR, GCS, and BR, respectively, and added 5 additional comprehensive variables: number of body regions (NBR), age, gender, injury mechanism, and hospital fixed effects. Simultaneously, the optimal ratio of GCS and BR death probability was used to modify traumatic death probability (TDP) of TMR. We applied average of the first 3 highest (worst) TDP values as WADP for each AIS predot code (Appendix B).

Of the total data set (not used for the estimation of WADP values), 20% was used to estimate IMP. To calculate IMP coefficient according to logistic regression model (Table 4), and deduce specific formula for the IMP (Appendix C). The remaining 20% of the data (validation data set) was not used for the estimation of WADP values or the development of IMP to estimate the statistical performance of IMP.

### 2.3 Customization of each trauma model

This validation data set enables us to test the performance of the TMPM, single worst injury (SWI) and IMP. TMPM model applies the original MARC values, which are sorted according to the severity in multiple injury patients, and determines whether the 2 worst injury in the same BR, and then calculates each TMPM probability of death. An SWI model is defined as the WADP value for the worst injury (i.e., the greatest WADP value). IMP contains 5 of the worst (max) WADP values of the injuries in severity order, determines whether or not the worst and second worst injuries are in the same BR, the product of the 2 worst injuries WADP values is a variable and NBR (as ln(NBR) and NBR^0.382^, fractional polynomial analysis is suggested^[12]^) in each individual injury patient, according to the IMP-specific formula to calculate the probability of death. Meanwhile, all 3 models are then re-estimated after adding age, gender, and injury mechanism to simple injury models, which only include the information on anatomic injury. We apply the robust variance estimators,^[13]^ because the outcomes of patients treated at the same trauma center may be correlated.

### 2.4 Statistical analysis

The statistical performance of the trauma models was assessed with the area under the receiver operating characteristic curve (ROC), the Hosmer–Lemeshow (HL) statistics, and the Akaike information criterion (AIC). The AIC is a measure of the Kullback–Leibler information number, which quantifies how close a statistical model approaches the true distribution. The reason of comparison is that the best model in a particular data set is the model with the lowest AIC. A bootstrapping algorithm (1000 replications) was used to calculate the bias-corrected 95% confidence intervals for the ROC and the HL. A *P* < .05 was considered statistically significant. All statistical analyses were performed with STATA/SE version 12.0 for Windows. The study was approved by the Institutional Review Board of Hangzhou Normal University, People's Republic of China.

## 3 Results

In this study, the total of the WADP is 1329 different AIS coded injuries (Supplemental Digital Content 1.xls). These WADP values range from 0.008 for a minor injury (AIS 730299.1: “digital nerve injury no further specify [NFS]”) to a value of 3.687 for an unsurvivable injury (AIS 919208.6: “burn of larynx trachea and lung”). WADP values provide finer precision than the 6 consecutive integer values useable to the AIS severity. It is interesting to note that seemingly “minor” injuries (e.g., AIS 341402.1: “sprain/strain thyroid region”) are assigned to higher WADP values, while others are “severe” injuries (e.g., AIS 311000.6: “open wound of neck NFS complicated”) assigned WADP values are relatively low. We think that they are appropriate, because by design, WADP values reflect an injury's propensity lead to death rather than their subjective severity.

Patient demographics are summarized in Table 1. In this study, the mortality rate was 2.76%, of which White and Black were 66.2% and 14.3%, respectively. The 2 most common causes of trauma were low falls (38.6%) and motor vehicle accidents (36.2%). Females accounted for 35.5%.

**Table 1:**
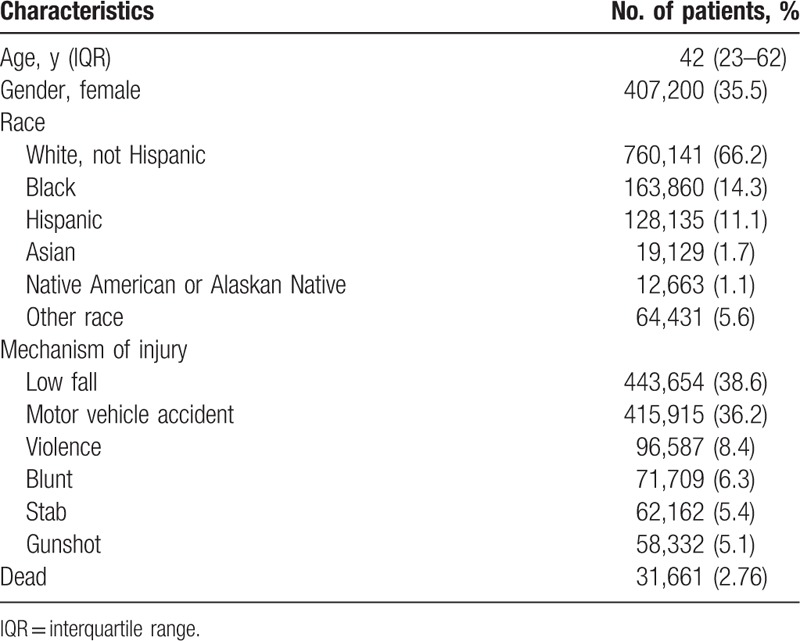
Patients’ demographics.

The statistical performance of all of the models is shown in Tables 2 and 3. The IMP exhibits significantly both better discrimination, calibration, and the AIC statistic, compared with either the TMPM or the SWI model. With the addition of age, gender, and mechanism of injury, IMP continued to exhibited superior model performance, compared with TMPM or the SWI model. The IMP coefficients are shown in Table 4.

**Table 2:**
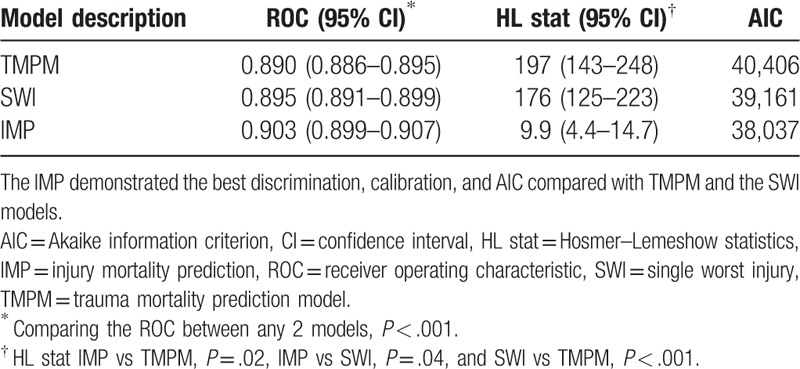
Model performance: anatomic injury models.

**Table 3:**
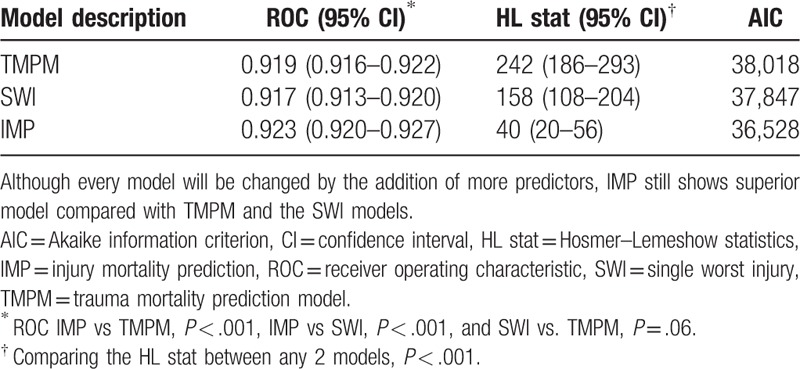
Model performance: anatomic injury models augmented with age, gender, and mechanism of injury.

**Table 4:**
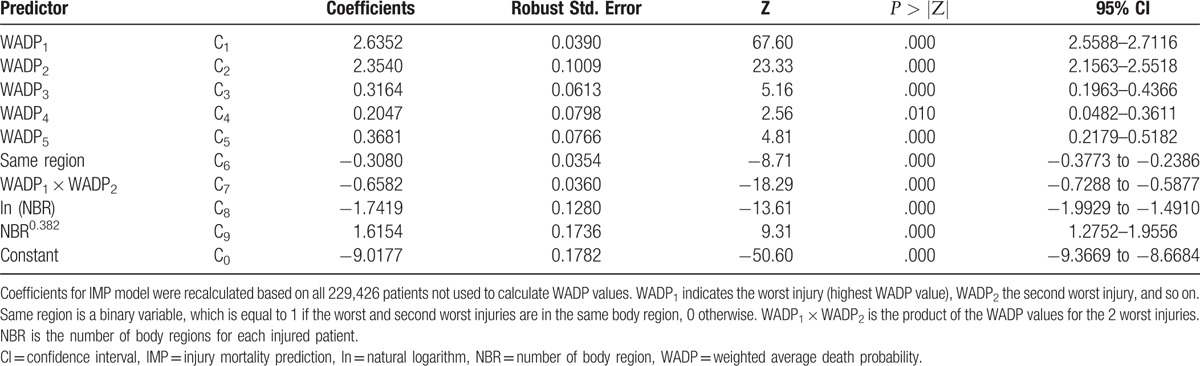
IMP regression coefficients.

**Figure 2:**
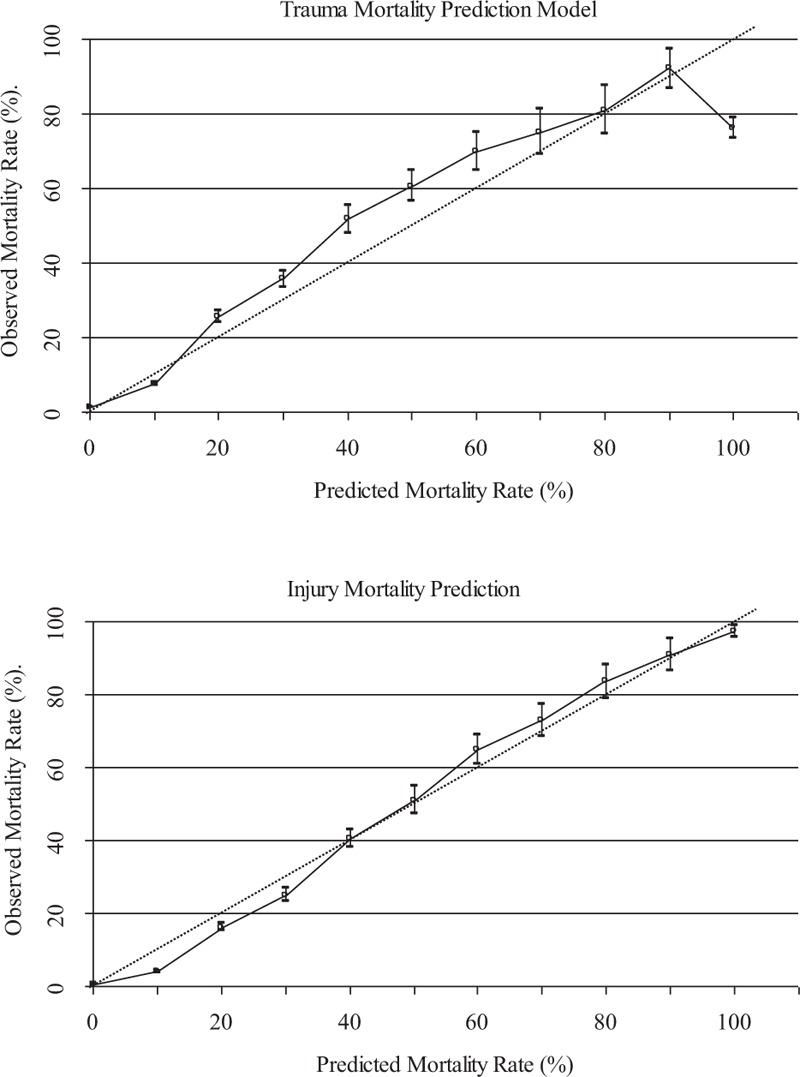
Solid lines indicate mortality rates for different TMPM and IMP values. The dotted reference lines represent perfect calibration. 95% Binomial confidence intervals for 2 models are based on the same validation dataset of 229,895 patients. IMP = injury mortality prediction, TMPM = trauma mortality prediction model.

Figure 2 shows the TMPM and IMP, respectively, against the actual mortality rates. The TMPM mortality rates were distributed at 2-sided dotted reference line but displayed an arch shaped curve. The data fits a linear function with an *R*^2^ = 0.9134, *P* < .001. The IMP mortality rates were uniformly distributed much closer to the dotted reference line and the data fits a linear function with an *R*^2^ = 0.9913, *P* < .001.

## 4 Discussion

With the continuous improvement of the diagnosis and treatment, trauma mortality will also significantly decrease. Although patients with injuries treated at trauma centers have better outcomes compared with patients admitted to nontrauma centers,^[14]^ there are still significant differences in the outcomes among Level I trauma centers for severity of similar trauma patients,^[15]^ which may result from the inaccuracy of the current scoring systems.

Designing a perfect trauma scoring system to accurately predict mortality and outcome is a problem remaining to be solved by trauma surgery experts. It is necessary that has reliable large data set and a feasible calculation method to solve this problem. The NTDB has the largest and the most credible trauma dataset around the world. It includes trauma data for different trauma centers in different regions of the United States and contains information that provides us with research. Currently, TMPM is the best trauma score method, and its ability is better than the ISS and NISS in predicting mortality.^[8]^ Thus, this research only compared IMP with TMPM rather than ISS and NISS.

In this TMR and WADP development data set, TMR value is set to 0.8 × 0.618 (in reference to population crude death rate of approximately 0.8% in the United States during the year 2010 and 2011),^[16]^ take the golden point 0.618. Because the average age of the injured patients was younger, then, actual death rate for different AIS predot codes was 0, will increase death cases apparently. However, in fact, its impact on the whole validation data set can be negligible. There are 11 (only contains 15 patients) single or multiple injuries with 100% mortality, but these single or multiple injuries each has less cases, and the majority of code pairs of 100% mortality have only 1 case. In this study, a case survivor was added, then we calculated TMR value (Appendix A), which seems to decrease death cases. In fact, this method of correction is appropriate, and in more accord with clinical practice.

In this study, specific AIS predot code for different individual patients is used, 3 separate logistic regression models were created using TMR, GCS, and BR. Meanwhile the optimal proportion of GCS and BR death probability was used to correct TDP of TMR, in order to achieve the best value. This method combines rigorous statistical regression models with mathematical properties in order to improve the prediction accuracy. For specific AIS predot code using different individual patients, the 3 highest TDP weighted as its final average value (i.e., WADP) (Appendix B). For each individual, the contribution to the death depends primarily on the 3 most serious injuries, such as ISS, NISS, LISS, etc.

The coefficients for the worst injuries were approximately 3 times the coefficients for the less severe injuries (results not shown) in this study, when the 2 worst injuries were not interacted in IMP. This indicates that the worst injury determine the probability of death of individual injury patients in a certain extent. Therefore, SWI models in the prediction of death are also efficient.^[17]^ The TMPM holds that a patient's 5 worst injuries determine the possibility of mortality to a great extent.^[7]^ TMPM is more than ISS in the number of injured regions. ISS allows calculation at the 3 best injuries.^[4]^ The study considers the sum of the 5 most severe WADP values as IMP value because only 5 coefficients of the worst injuries for each individual patient were statistically significant in our data set. We find that the discrimination and calibration of the IMP are slightly better than TMPM which is statistically significant (Table 2).

This study finds that the NBR for each trauma patient has an inherent and useful parameter in the prediction ability of trauma death. NBR is better than patient's age or gender in both the discrimination and the relevance (results not shown). However, the existing scoring methods (such as ISS, LISS, TMPM, etc.) do not cover it. In this study, NBR is added to improve IMP predict trauma results.

Generally speaking, the addition of any other information (e.g., systolic blood pressure, respiratory rate, GCS, etc.) always improves the accuracy of predictions.^[7,18]^ The basic IMP is attractive because only anatomical injury information is available. However, IMP can also serve as the solid foundation for the addition of more sophisticated prediction information (e.g., physiological parameters) to further improve the accuracy of the prediction results. There is similar result in this study, the addition of GCS score can improve the ROC of the IMP from 0.923 to 0.943 (including 217,480 patients with GCS score, this is not shown in analysis). When we added age, gender, and mechanism of injury, the IMP had better discrimination, calibration, and AIC than the TMPM and SWI with the additional information (Table 3).

In this study, as required by the trauma score method, we have removed all cases related to patients with single or multiple injuries that only contains AIS severity code of 9. Nevertheless, there are still BRs where AIS severity code equals to 9 in multiple injuries cases (a total of 22 [1.66%] AIS predot codes, involving 0.61% of the BRs), and their corresponding WADP values could be calculated. However, the impact to the entire data set can be neglected.

This study only applies AIS-1998 rather than AIS-2005 version. Although AIS-2005 is more sophisticated and may be better in predicting trauma mortality than AIS-1998.^[2,19,20]^ However, if we want to compare the differences between 2 versions, we can detect when there are quite a few cases in the future.

The IMP prediction of mortality is based solely on the WADP of patients’ AIS predot codes. We believe that clinicians can calculate the value of an injury score. The popularity of any injury score methods to some extent dues to its easiness of calculation. IMP inherits and extends this advantage, and relies on the AIS predot codes for each injury. Meanwhile, the IMP directly expresses the probability of death, which is similar to TMPM.^[7]^

## 5 Limitations

This study uses NTDB data and inherits its limitations. It does not contain all hospitals (trauma and nontrauma centers) trauma patients and is not population based. TMR values are only set as reference of traumatic death. They require computation of WADP values by combining with the logistic regression model and mathematical characteristics. The possibility of death in patients is then evaluated with different AIS predot codes. The calculation method is unique in this study. Although the calculation process is somewhat complicated, it can improve the ability of death prediction. Simultaneously, when WADP values are applied to other trauma data sets, over time these values may be changing, perhaps as the outcome of new therapies. The WADP values, however, are that they are simple periodically to recalculate WADP values based on the latest data set.

## 6 Conclusions

The IMP has slight improvement in discrimination and calibration compared with the TMPM and can accurate predictions of mortality than it. Therefore, we consider it as a new feasible scoring method in trauma research.

## Supplementary Material

Supplemental Digital Content

## Supplementary Material

Supplemental Digital Content
