# Supplementary material for: An injury mortality prediction based on the anatomic injury scale
Source: Medicine (Baltimore). 2017 Sep 1;96(35):e7945. doi: 10.1097/MD.0000000000007945 (PMC5585518; doi:10.1097/MD.0000000000007945)
Supplement: Supplemental Digital Content [file medi-96-e7945-s002.doc]

**Appendix A**

**The Calculating Method of the TMR**  First, for the calculation of a single trauma mortality rate (SMR). In single injury cases, specific AIS predot code deaths (D1) divided by the total number of cases (T1) and then multiplied by 100, which is SMR = D1 / T1 × 100. If D1 = 0, we assume 0.8% of mortality multiplied by 0.618, then divided by the total number of cases (T1) as a trauma mortality rate, SMR = 0.8 × 0.618 / T1. If D1 = T1, we assume to added the cases with one survivor, SMR = D1 / (T1+1) × 100.

Second, for the calculation of multiple trauma mortality rate (MMR) (In this study multiple injury patients have an average of 4.4727 injuries per patient). In multiple injury cases, specific AIS predot code deaths (D2) divided by the total number of incidents (T2) multiplied by 100 and then divided by 4.4727, MMR = D2 / T2 × 100 / (4.4727 × 0.618). If D2 = 0, we assume 0.8% of mortality divided by the product of 4.4727 and the total number of cases (T2), MMR = 0.8 / (T2 × 4.4727). If D2 = T2, we assume to added the cases with one survivor, MMR = D2 / (T2+1) × 100 / (4.4727× 0.618).

Third, merge single and multiple trauma mortality rates of specific AIS predot code as total trauma mortality rate (TMR), TMR = (SMR × T1 + MMR × T2) / (T1 + T2). In order to ensure that each TMR is different, there is same TMR value in different AIS predot codes, which reduce the 0.00001 in turn according to the size of the sum of T1 and T2 and different AIS predot codes in this study, a total of 1329 TMR values for different AIS codes are listed in the [SDC 1](../8.%20SDC%201.xls).xls.

TMR indicates the trauma mortality rate with specific AIS predot code. SMR indicates the mortality rate of single trauma with specific AIS predot code. MMR indicates the mortality rate of trauma incident with specific AIS predot code in multiple traumas, D1 indicates the number of death cases of a single injury with specific AIS predot code. D2 indicates the number of death incidents of multiple injury with specific AIS predot code. T1 indicates the total number of single trauma cases with specific AIS predot code. T2 indicates the total number of incidents with specific AIS predot code in multiple traumas. In year 2010 and 2011, the population crude death rate in the United States is 0.8%. [^16^](#OLE_LINK16) This study introduces the golden ratio, 0.618, as a parameter.

**Appendix B**

**Calculating WADP values**

Each AIS predot code is replaced by its TMR value ([SDC 1](../8.%20SDC%201.xls)). This study mainly used 1329 TMR values of possible injury AIS predot codes, their corresponding body regions (BR) and GCS as fundamental predictors to create three separate regression models, respectively. 5 additional variables were included in three models to decrease variance: number of body region (NBR), age [as age^3^ and age^3^ × log(age), fractional polynomial analysis is suggested here [^12^](#OLE_LINK12)], gender, injury mechanism and independent influence of hospital. [*P*_TMR_ = *P*(death)1, *P*_GCS_ = *P*(death)2 and *P*_BR_ = *P*(death)3]. Mathematical expressions were as follows:

$$\begin{matrix} P(death)1=\Phi\left(\alpha_{0}+\alpha_{1}\sum_{i=1}^{1329} TMR_{i}+\alpha_{2}NBR+\alpha_{3}age^{3}+\alpha_{4}(age^{3})\log(age)\right. \\ \left.+\alpha_{5}gender+\alpha_{6}\sum_{r=1}^{6} mechanism_{r}+\alpha_{7}\sum_{s=1}^{476} H_{s}\right) \end{matrix}$$

$$\begin{matrix} P(death)2=\Phi\left(\beta_{0}+\beta_{1}\sum_{j=3}^{15} (16-GCS_{j})+\beta_{2}NBR+\beta_{3}age^{3}+\beta_{4}(age^{3})\log(age)\right. \\ +\left.\beta_{5}gender+\beta_{6}\sum_{r=1}^{6} mechanism_{r}+\beta_{7}\sum_{s=1}^{476} H_{s}\right) \end{matrix}$$

$$\begin{matrix} P(death)3=\Phi\left(\gamma_{0}+\gamma_{1}\sum_{k=1}^{9} BR_{k}+\gamma_{2}NBR+\gamma_{3}age^{3}+\gamma_{4}(age^{3})\log(age)\right. \\ \left.+\gamma_{5}gender+\gamma_{6}\sum_{r=1}^{6} mechanism_{r}+\gamma_{7}\sum_{s=1}^{476} H_{s}\right) \end{matrix}$$

where *P*(death)1, *P*(death)2 and *P*(death)3 are the mortality predicted by the *P*_TMR_, *P*_GCS_, and *P*_BR_, respectively and Φ is the standard normal cumulative distribution function.

TMR*_i_*, *i* = 1, ..., 1329 is a binary indicator variable for each of the 1329 modified AIS predot codes.

GCS*_j_*, *j* = 3, ..., 15 is a binary indicator variable for each of the 13 GCS values.

BR*_k_*, *k* = 1, ..., 9 is a binary indicator variable for each of the 9 body region codes.

mechanism*_r_*, *r* = 1, ..., 6 represents a binary indicator variable for the mechanism of injury.

H*_s_*, *s* =1, ..., 476 is a binary indicator variable for each of the 476 hospitals.

We then combined five variables: *P*_TMR_, *P*_GCS_, *P*_BR_, AIS and TMR, to estimate each traumatic death probability (TDP) value:

$$\begin{matrix} TDP=\ln\left(\ln\left(\frac{P_{TMR}+6.2297}{TMR}+1\right)\times\right.\left(\ln\left(\frac{P_{GCS}+6.6044}{TMR}+1\right)/\ln\left(\frac{P_{BR}+6.7364}{TMR}+1\right)\right)^{1.618} \\ \left.\times TMR\times\ln\left(\frac{AIS^{3}}{2}+1\right)^{0.618/2}\times\ln\left(\frac{TRN^{3}}{2}+1\right)^{0.382/2}+1\right) \end{matrix}$$

where “ln” indicates natural logarithm. TMR round number (TRN) = TMR^0.5^ + 0.5 (Round numbers). The TMR values are transformed into 6 integer values mathematically, their values are similar to the AIS severity codes. AIS is an AIS severity code that ranges from 1 to 6. If AIS severity code is 9, the above method is applied as well. Therefore, the AIS = TRN. In order to ensure that each variable is not negative, corresponding constants (6.2297, 6.6044 and 6.7364) for *P*_TMR_, *P*_GCS_ and *P*_BR_ are added respectively.

This study is to calculate the weighted average of the three most serious (highest) TDP values for each injury, namely, Weighted Average Death Probability (WADP). Calculation method is as follow:

$$WADP_{j}=\frac{1}{N_{j}}\sum_{i=1}^{N_{j}} TDP_{i}$$

where N_j_ is the number of specific AIS predot code for the three worst (highest) TDP values or whatever are lesser than the third most serious TDP value.

**Appendix C**

**Estimating IMP Value**

It is necessary to replace AIS codes with their respective WADP values ([SDC 1](../8.%20SDC%201.xls)). WADP values are sorted by the severity among patients who sustained multiple injuries. We calculate the coefficient values of IMP with the logistic regression method. Following formula is deduced to assess the IMP probability of death for an individual patient:

$$P\left(death\right)=\Phi\left(C_{0}+\sum_{i=1}^{5} C_{i}\times I_{i}+C_{6}\times S+C_{7}\times I_{1}\times I_{2}+C_{8}\times ln\left(NBR\right)+C_{9}\times NBR^{0.382}\right)$$

where Φ is the standard normal cumulative distribution function, and *I*_1_, ..., *I*_5_ are the WADP values for the five worst injuries, ordered with the greatest WADP value (worst injury) first, the second greatest WADP value second, up to the fifth worst injury. *S* is an indicator variable equal to 1 if the two worst injuries are in the same body region (i.e., the two worst injuries AIS coding scheme have the same first digit), 0 otherwise. *I*_1_×*I*_2_ represents the product of the WADP values for the two worst injuries. NBR is the number of body regions in an individual injured patient. “ln” indicates natural logarithm. *C*_0_, ..., *C*_9_ are coefficients in Table 4.

The authors will provide an IMP calculator available to the Visual Foxpro 6.0 platform. In fact, the IMP can also be calculated by any computer language.
